# Interactive Sonification of Spontaneous Movement of Children—Cross-Modal Mapping and the Perception of Body Movement Qualities through Sound

**DOI:** 10.3389/fnins.2016.00521

**Published:** 2016-11-11

**Authors:** Emma Frid, Roberto Bresin, Paolo Alborno, Ludvig Elblaus

**Affiliations:** ^1^Sound and Music Computing, Media Technology and Interaction Design, School of Computer Science and Communication, KTH Royal Institute of TechnologyStockholm, Sweden; ^2^Casa Paganini - Infomus Research Centre, DIBRIS Dipartimento di Informatica, Bioingegneria, Robotica e Ingegneria, Università di GenovaGenova, Italy

**Keywords:** interactive sonification, movement analysis, movement sonification, mapping, motion capture, perception

## Abstract

In this paper we present three studies focusing on the effect of different sound models in interactive sonification of bodily movement. We hypothesized that a sound model characterized by continuous smooth sounds would be associated with other movement characteristics than a model characterized by abrupt variation in amplitude and that these associations could be reflected in spontaneous movement characteristics. Three subsequent studies were conducted to investigate the relationship between properties of bodily movement and sound: (1) a motion capture experiment involving interactive sonification of a group of children spontaneously moving in a room, (2) an experiment involving perceptual ratings of sonified movement data and (3) an experiment involving matching between sonified movements and their visualizations in the form of abstract drawings. In (1) we used a system constituting of 17 IR cameras tracking passive reflective markers. The head positions in the horizontal plane of 3–4 children were simultaneously tracked and sonified, producing 3–4 sound sources spatially displayed through an 8-channel loudspeaker system. We analyzed children's spontaneous movement in terms of energy-, smoothness- and directness-index. Despite large inter-participant variability and group-specific effects caused by interaction among children when engaging in the spontaneous movement task, we found a small but significant effect of sound model. Results from (2) indicate that different sound models can be rated differently on a set of motion-related perceptual scales (e.g., expressivity and fluidity). Also, results imply that audio-only stimuli can evoke stronger perceived properties of movement (e.g., energetic, impulsive) than stimuli involving both audio and video representations. Findings in (3) suggest that sounds portraying bodily movement can be represented using abstract drawings in a meaningful way. We argue that the results from these studies support the existence of a cross-modal mapping of body motion qualities from bodily movement to sounds. Sound can be translated and understood from bodily motion, conveyed through sound visualizations in the shape of drawings and translated back from sound visualizations to audio. The work underlines the potential of using interactive sonification to communicate high-level features of human movement data.

## 1. Introduction

Interactive sonification is the discipline of interactive representation of data and data relationships by means of sound. If properly designed, it serves as a powerful and effective information display. In order to successfully design sonification applications one has to consider how meaning is ascribed to certain sounds. Closely linked to this topic is the notion of *mapping*, i.e., how input parameters are mapped to auditory output parameters in order to convey properties of the data through perceptually relevant acoustic features. There is a large set of possible mappings that could be used within the context of sonification of human movement (see e.g., Dubus and Bresin, 2013 for an overview). However, only a small subset of these mappings will produce perceptually relevant results (Roddy and Furlong, 2014). Our work is motivated by the fact that if links between sound properties and movement can be found, design of auditory information displays and sonification applications could be improved through use of more perceptually relevant and intuitive mappings. The work presented in this paper serves as first investigation in a series of attempts aimed at finding perceptually relevant attributes of sound synthesis for sonification of human movement. The aim is to investigate if different sound models can evoke different associations to motion and thereby induce different spontaneous movement characteristics.

It is clear that musical sounds can induce human body movement, but can certain properties of a sound influence, and be associated to, specific properties of bodily movement? The notion of *embodied cognition* assumes that the body is involved in, and required for, cognitive processes (Lakoff and Johnson, 1980, 1999). Following an embodied cognition perspective, we can approach music by linking perception to our body movement (Leman, 2007). Bodily movements can thus be said to reflect, imitate or support understanding of the content and structure of music (Burger et al., 2013). In our study we aim to expand on this notion of a link between music and motion to non-musical sounds. Based on the notion that music carries the capacity to activate the embodied domain of sounds by inducing movement (see for example Zentner and Eerola, 2010), we assume that spontaneous bodily movement to interactive sonification may reflect and imitate aspects of sound produced by the sonification system.

Following the ecological approach to auditory perception (Gaver, 1993) and the notion that no sound is produced without movement, we formulate the hypothesis that a sonfication mode characterized by a sustained and continuous amplitude envelope will be associated to properties related to smooth, continuous movements. A non-continuous sound characterized by acoustically abrupt events should accordingly be associated to properties related to non-continuous movements. We designed two sound models based on these hypotheses: one continuous sound model and one sound model characterized by a high level of amplitude modulation and sudden amplitude irregularities. For comparative purposes, we also designed a model that was considered to be perceptually in between these two models in terms of irregularities in amplitude.

The three sound models were used in three different experiments in which we investigated the relationship between above mentioned properties of a sound and bodily movements. Study 1 focused on investigating if three sound models would evoke different movement characteristics among children when moving freely in a room. Assuming that spontaneous movement in an interactive sonification task can be understood as a means of exploring the presented sound, our hypothesis was that movement at a specific point of measurement could be influenced by the specific sound model used at that time point. Study 2 focused on investigating if sound models used in 1 were rated differently on a set of motion-related perceptual scales by another group of participants. Our hypothesis was that different sound models would be rated differently. Study 3 focused on investigating if drawings depicting sounds recorded in Study 1 could be easily identified and matched to respective sound model in a forced-choice experiment. We hypothesized that participants would be able to correctly match recordings of one sound model to an abstract visual representation, i.e., a sound visualization in the form of a drawing, of the same sound model.

## 2. Background

### 2.1. Sound and movement

The link between sound and movement has been investigated in numerous studies throughout the years. Following an ecological perception point of view, interpretation of sounds is founded on knowledge on gestural actions required to produced the sound in question (Gaver, 1993). Keller and Rieger (2009) found that simply listening to music can induce movement. Janata and Grafton (2003) showed that passive music listening can involve activation of brain regions concerned with movement. As stated by Godøy and Jensenius (2009), it is not far-fetched to suggest that listeners' music-related movements often match overall motion and emotional features of a musical sound: guidelines for traditional gestures for musical conductors state that legato elicits smooth, connected gestures, while accented and rhythmic music elicits shorter and more jerky movements (Blatter, 2007).

A couple of studies have focused on spontaneous movement to musical sounds and how such movement trajectories can be analyzed and classified (Casciato et al., 2005; Godøy et al., 2005; Haga, 2008). Up to this point, few studies have focused on motion analysis of children's spontaneous movement patterns to sound and music (see for example Zentner and Eerola, 2010), especially in the context of interactive sonification (e.g., Källblad et al., 2008). The topic of spontaneous movement to musical sounds is related to research on *music-induced movement*, where focus lies on how people react corporeally to music. Several factors, such as musical features and individual factors, can affect the characteristics of such music-induced movements (Burger et al., 2013). Leman (2007) defined three components that could influence corporeal articulations in music-induced movement: *synchronisation, embodied attuning* and *empathy*. *Synchronisation* is a fundamental component that deals with synchronisation to a beat; *Embodied attuning* concerns the linkage between body movements to musical features more complex than a basic beat (such as e.g., harmony, melody, rhythm, tonality and timbre); *Empathy* links musical features to emotions and expressivity. According to Leman (2007), spontaneous movements to music appear to be closely related to predictions of local bursts of energy in the audio stream, such as beat and rhythms. Although multiple studies have focused on the effect of synchonisation/beat (Toiviainen et al., 2009; Burger et al., 2014) and expressive features in music (Buhmann et al., 2016) in this context, rather few projects have focused on the effect of more complex musical features such as e.g., timbral properties and their effect on music-induced spontaneous movement (see for example Burger et al., 2013).

There are some examples of studies on the relationship between sound and spontaneous movement in which participants have been instructed to trace sounds that they hear, i.e., to trace the perceptual features of a sound (see for e.g., Godøy et al., 2006, 2010; Nymoen et al., 2010). This is usually referred to as “*sound-tracing”*, a concept first introduced by Godøy et al. (2005) which is defined as the process of rendering perceptual features of sound through body motion. In a study by Caramiaux et al. (2014) in which participants were instructed to synchronously perform a gesture to a sound, it was found that if the cause of a sound could be identified, participants would perform spontaneous gestures that attempted to mimic the action producing the sound. However, if the sound contained no perceivable causality, the spontaneous movement would trace contours related to acoustic features. Moreover, abstract sounds were found to result in less gesture variability.

In the study presented in this paper, we use drawings as a means of describing perceived sounds. Drawings can, similar to words and gestures, serve as a high-level approach to description. Drawings have previously been used in contemporary music to either compose or describe music (Thiebaut et al., 2008). The association between sound and shapes has been investigated in numerous studies throughout the years in research on what is usually referred to as *shape symbolism*. Shape symbolism is a family of multisensory phenomena in which shapes give rise to experiences in different sensory modalities, the most common example being the “*Bouba-Kiki effect”* (Kohler, 1929, 1947), in which the words “kiki/takete” and “bouba/maluma” are associated with angular vs. rounded shapes. The hypothesis in our study, as previously suggested by Merer et al. (2013), is that drawings are a relevant means of describing motion in an intuitive way, and that the use of abstract sounds, in which the physical sources can not be easily identified, provide relevant and unbiased keys for investigating the concepts of motion.

### 2.2. Movement analysis

In order to analyze movements of the children participating in the motion capture experiment described in Section 3, we extracted motion features from motion capture recordings. We followed the multi-layered conceptual framework for the analysis of expressive gestures proposed by Camurri et al. (2016). This framework consists of four layers allowing for both a bottom-up (from Layer 1 to 4) and top-down (from Layer 4 to 1) analysis; Layer 1 - Physical signals (e.g., positional data captured by IR cameras), Layer 2 - Low-level features (e.g., velocity), Layer 3 - Mid-level features (e.g., smoothness), Layer 4 - Expressive qualities (e.g., emotion). Following this layered approach, we included low-level features (i.e., Energy Index, *EI*, and Smoothness Index, *SI*, of head movements) and one mid-level feature (i.e., Directness Index, *DI*). The above mentioned features have previously been used in different contexts and research purposes in order to describe the expression of human gestures (Camurri et al., 2002), for investigating the emotional mechanisms underlying expressiveness in music performances (Castellano et al., 2008) and as potential descriptors to infer the affective state of children with Autism Spectrum Condition (Piana et al., 2013). We decided to include the Energy Index (*EI*) in the investigation since we hypothesized that this feature could be highly correlated with properties of the movements elicited by different sound models. Moreover, we decided to include Smoothness- and Directness-Index (*SI* and *DI*), since we were interested in how continuous the movement trajectories of the children would be for different sound models. A description of each feature can be found below.

#### 2.2.1. Energy index

This feature concerns the overall energy spent by the user during a movement and is computed as the total amount of displacement in all of the tracked points. Given a two-dimensional tracking information, we can define velocity of the *i*-th tracked point at frame *f* as:

(1) $$v_{i}(f)=\sqrt{\left({\dot{x}}_{i}{\left(f\right)}^{2}+{\dot{y}}_{i}{\left(f\right)}^{2}\right)}$$

Where ẋ_*i*_ and ẏ_*i*_ are the first derivatives of the position coordinates. The Energy Index *EI* can then be computed as:

(2) $$\begin{array}{l} EI\left(f\right)=\frac{1}{2}\overset{J}{\underset{i=1}{\sum }}m_{i}\cdot v_{i}^{2}\left(f\right), \end{array}$$

where *J* is the number of tracked points (or joints) of the subject's body. In the context of our experimental setup, we computed the energy of each subject by tracking only their head movements, using a single rigid body (a combination of markers in a unique pattern that could be identified by the tracking system). *EI*(*f*) is therefore an approximation of the head's kinematic energy estimated as one single point's kinetic energy (max number of points *J* = 1). To simplify the calculation, the weight *m*_1_ was also set to 1.

#### 2.2.2. Smoothness index

The mathematical concept of smoothness is associated to the rate of variation of a function waveform. A smooth function varies “slowly” over time; smooth functions belong to the *C*^∞^ class, i.e., functions that can be derived an infinite number of times. The third derivative of the movement position has often been used as descriptor for the smoothness of a motion trajectory (Flash and Hogan, 1985). Our algorithm for computing smoothness is based on the studies made by Viviani and Terzuolo (1982) and Todorov and Jordan (1998) that show an existing correlation between trajectory curvature and velocity. The Smoothness Index *SI* can be computed from the trajectory curvature and velocity. The curvature *k* measures the rate at which a tangent vector to the trajectory curve changes as the trajectory bends. As an example, the trajectory of a rigid body following the contour of a geometric shape, such as a square, will bend sharply in some points. This trajectory will thus be characterized by high curvature and low smoothness. In contrast, a straight line trajectory will have zero curvature and infinite smoothness (Glowinski et al., 2011).

We can define a bi-dimensional trajectory consisting of collection of consecutive coordinates *x*_*i*_(*f*) and *y*_*i*_(*f*) of the *i*-th tracked joint at frame *f* (in our case max *i* corresponds to one tracked point) and its velocity *v*_*i*_(*f*). The trajectory curvature *k* can be computed as:

(3) $$k(x_{i}(f),y_{i}(f))=\frac{{\dot{x}}_{i}(f)\cdot {\ddot{y}}_{i}(f)-{\dot{y}}_{i}(f)\cdot {\ddot{x}}_{i}(f)}{\left(\sqrt{{{\dot{x}}_{i}}^{2}(f)+{{\dot{y}}_{i}}^{2}(f)}\right){\;}^{3/2}},$$

where ${\dot{x}}_{i}(f)$, ${\dot{y}}_{i}(f)$, $\ddot{x_{i}}\left(f\right)$ and $\ddot{y_{i}}\left(f\right)$ are the first- and second-order derivatives of the coordinates *x* and *y*. In our work we define the Smoothness Index *SI* as the Pearson correlation coefficient ρ computed on the quantities *log*(*k*) and *log*(*v*). This index gives a measure of the relationship between velocity and curvature and it is calculated as:

(4) $$\begin{array}{l} \rho \left(k,v\right)=\frac{cov\left[\log\left(k\right),\log\left(v\right)\right]}{\sigma_{\log\left(k\right)}\cdot \sigma_{\log\left(v\right)}} \end{array}$$

In the calculus of the Smoothness Index *SI*, *k* and *v* are evaluated over short time windows (30 ms). Therefore, we could approximate the covariance *cov*[*log*(*k*), *log*(*v*)] by 1, as the *k* and *v* variate (or not) approximately at the same rate. We can then simplify the definition of the Smoothness Index *SI* to:

(5) $$\begin{array}{l} SI=\rho \left(k,v\right)=\frac{1}{\sigma_{\log\left(k\right)}\cdot \sigma_{\log\left(v\right)}} \end{array}$$

#### 2.2.3. Directness index

The Directness Index *DI* is a measure of how much a given trajectory, generated by a tracked joint (in our case, point), is direct or flexible. *DI* has been detected as one of the main motion features in the process of recognizing emotions (De Meijer, 1989). A direct movement is characterized by almost rectilinear trajectories. The *DI* is computed as the ratio between the length of the straight line connecting the first and last point of a trajectory and the sum of the lengths of each segment constituting the trajectory itself. Therefore, the more the *DI* value is near to value 1, the more direct is the trajectory. In the case where we have a two-dimensional trajectory, the Directness Index *DI* can be computed as:

(6) $$DI=\frac{\sqrt{{\left(x_{end}-x_{start}\right)}^{2}+{\left(y_{end}-y_{start}\right)}^{2}}}{\sum_{i=k}^{N}\sqrt{{\left(x_{k+1}-x_{k}\right)}^{2}+{\left(y_{k+1}-y_{k}\right)}^{2}}},$$

where *x*_*start*_, *y*_*start*_ and *x*_*start*_, *y*_*start*_ are the coordinates of the trajectory's start- and end-points in the 2D space and *N* represents the length of the trajectory.

## 3. Study 1: motion capture experiment

### 3.1. Method

The first study focused on investigating if three sound models would evoke different movement characteristics among children when moving freely in a room. Our hypothesis was that the specific sound model used at a particular time point could influence spontaneous movement of the children at a specific point of measurement. To investigate this hypothesis, we carried out a repeated measures experiment in which longitudinal data of participants' movements was collected in a motion capture room fitted with an 8-channel loudspeaker system. For each participant, x- and y-position and velocity of rigid body markers (placed on the head) were tracked. The data was fed to a sonification software providing real-time feedback of the performed movements.

#### 3.1.1. Participants

Two pre-school classes (4–5 vs. 5–6 years) from a kindergarten in Stockholm participated in the experiment. However, children in the age group 4–5 years failed to follow instructions in the experiment and were therefore excluded from the analysis, giving a total of *n* = 11 participants (2 boys and 9 girls, age 5–6 years, mean = 5.36, SD = 0.5). The participants were divided into groups of 3–4 participants, with a total of 3 groups. Each group participated in two sessions of the experiment: one recording session in the morning and one in the afternoon. A teacher from the kindergarten was always present during each session. We decided to work with children based on the assumption that younger participants would act more spontaneous than adults in a task involving free movement (since spontaneous movement is an integral aspect of active play). Moreover, it has been found in a study by Temmerman (2000) that children tend to have positive attitudes toward activities that provide opportunity to move freely to music.

There was no need for ethics approval since neither of the experiments presented in this paper involved deception or stressful procedures^1^. The research presented no risk to harm participants. Parents were required to return signed consent forms in which they agreed to their child's participation in the study. The informed consent included information about the study and the task; the form was distributed to make an effort to enable children to understand, to the degree they are capable, what their participation in the research would involve. All parents consented to both participation and possible future publishing of photos taken during the experimental session.

#### 3.1.2. Equipment

The experiment was run at the Multimodal Interaction and Performance Laboratory (PMIL), dedicated to experiments involving motion capture and spatial audio, at KTH Royal Institute of Technology, Stockholm, Sweden. The experimental setup consisted of several different software and hardware systems that together formed a chain, starting with the motion capture system and ending with the generation and spatialization of the sound. The motion capture system used was an *Optitrack Prime 41*^2^ setup using 17 IR cameras tracking passive reflective markers. The frequency of acquisition was 180 frames per seconds (resolution 4.1 MP, latency 5.5 ms). The cameras were placed on a circle, at a height of 2.44 m, following the perimeter of the room (the room measured 5.30 × 6.20 m). The trackable area in which the children were instructed to move was a rectangular area measuring 4.66 × 5.40 m, marked using tape on the floor. The system was controlled by the *Optitrack Motive*^3^ software. While tracking and recording, the *Motive software* also streamed data over a local network to a second computer, using the *NatNet*^4^ streaming protocol. A custom piece of software, written in C++, was running on the second computer that received the incoming NatNet data stream. Data was visualized and some additional calculations were performed on this second computer, whereafter original data was packaged with the calculated secondary data and send forward using the *Open Sound Control* (OSC) format^5^.

The final part of the chain was a third computer that took care of logging, sound generation and spatialization. The logging application, also a custom C++ solution, took every incoming OSC-message, added a local time stamp and wrote it to disk. The rationale for the double logging was to ensure that any issues caused by network transmission problems could be identified by comparing the recorded motion capture data in the head of the processing chain with the resulting data that actually arrived at the sound-producing computer. For the audio, a *Max/MSP*^6^ patch was used to both generate and spatialize the audio, as well as automatically run through the set of sound models for each session in the experiment. The *Max/MSP* patch also reported every change of state in the experiment with an OSC message to the logging application, meaning that the switching between sound models was recorded together with the movement data. A regular digital video camera was used to record all experiment sessions. The camera was mounted on a tripod in a corner of the lab and was kept recording for the entire duration of the sessions.

After the experiment, motion capture data was pre-processed and segmented whereafter each segmented file was streamed via OSC to *EyesWeb* for feature extraction. The *EyesWeb XMI platform*^7^ is a development and prototyping software environment for both research purposes and interactive applications which provides a set of software modules for analysis of human movements and behavior (Camurri et al., 2003). In the present study we used *EyesWeb* libraries for analysis of 2D movement trajectories to extract expressive motion feature describing human movements both at a local temporal granularity (Energy Index), and at the level of entire movement unit (Smoothness- and Directness Index). A movement unit can be for example a single movement or a whole phrase. In this particular study, a movement unit is defined as a time window with a specific duration.

#### 3.1.3. Stimuli

Each participant group was presented with five different auditory conditions. These conditions consisted of the three different sound models S1–S3 (sonification models) and excerpts from two pieces of music M1 and M2 (M1: “*Piano Trio No. 1 in D Minor, Op. 49: II. Andante con moto tranquillo”* by Felix Mendelssohn, and M2: “*Le Carneval des Animaux: Final”* by Camille Saint-Saëns). The conditions S1, S2, and S3 were interactive in the sense that the children's movement affected the generated sound. The musical conditions M1 and M2 were not interactive; children's movement was not mapped to the sound.

The musical pieces for conditions M1 and M2 were chosen since they in previous studies had been found to elicit certain emotions (Västfjäll, 2002; Camurri et al., 2006). M1 has been found to communicate tenderness and we therefore decided that this piece would be appropriate for the introductory part of the experiment. The purpose of including the M1 condition was to let the children get acquainted with the task and start moving to sound. M1 could however also have been used as a control condition, for comparative purposes. M2 has been found to elicit happiness and was therefore included in order to reward the children after successfully completing the experimental task.

For the sonification conditions S1–S3, we opted for sound models based on filtered noise. This decision was based on previous studies indicating that sounds with rich spectral content have been found to be more appealing to children with disabilities than other sounds (Hansen et al., 2012) and that the sound of speed and acceleration can be ecologically represented using simplified sound models reminding of the sound of wind, as for example in the sonification of rowing actions (Dubus and Bresin, 2015). Three sound models based on filtered white noise were defined: one producing smooth, wind-like sounds (S1); one model producing somewhat less smooth sounds characterized by more abruptly interrupted amplitude envelopes (S2); and one producing very choppy and clicking sounds due to a high-level of interruptions in the amplitude envelope (S3).

For each sound model S1–S3, low-level movement parameters (velocity, and x- and y-position of the participant in the horizontal plane) were mapped to acoustic parameters. Mappings were chosen among the most frequently used ones in previous research; for example location to spatialization, velocity to pitch and energy to loudness (see Dubus and Bresin, 2013, for a complete review of mappings). During the experiment, each child represented a sound source. Spatialization was done in such a manner that each child could hear the sound source follow his or her movement in the room. Therefore, there were up to four sounds generated simultaneously, representing the movements of four children. This was achieved through the use of a VBap 1.0.3 object (Pulkki, 1997) by mapping distance from the center point in the room to spread of the virtual sound source and by mapping the participant's angle from the center point to the azimuth angle.

**Figure 1:**
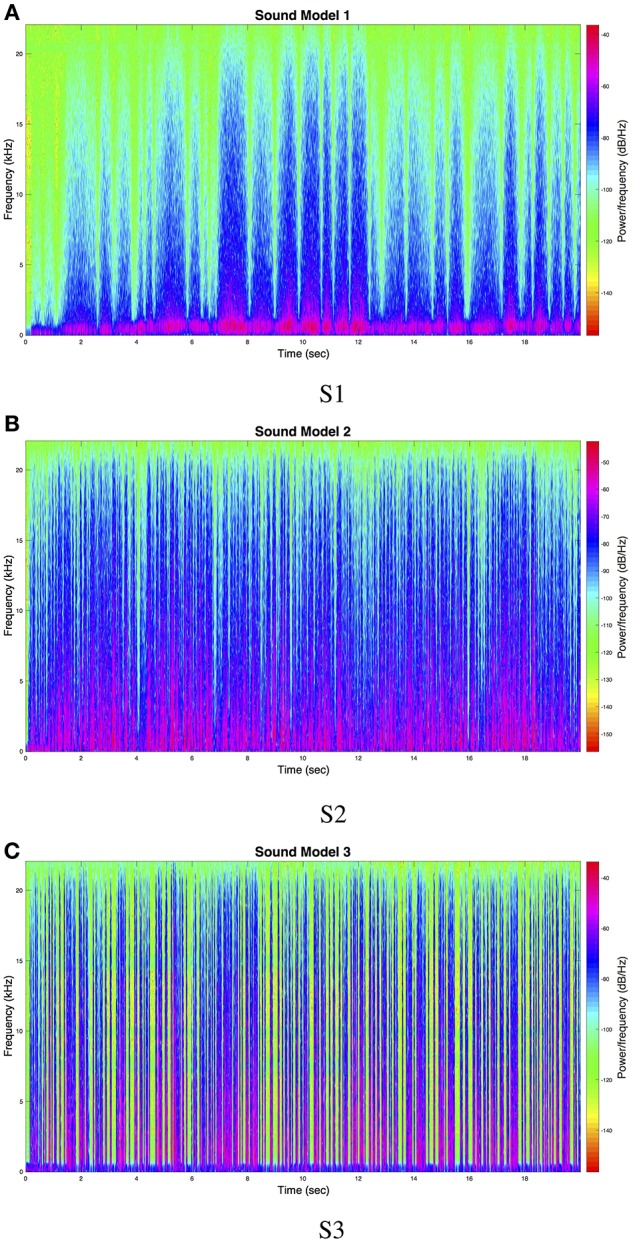
**Spectral content of 20 s of sound models (A)** S1, **(B)** S2, and **(C)** S3.

Sound model 1 (S1) was achieved by filtering white noise using the MaxMSP resonance filter biquad~ object with mode “resonant”. Velocity magnitude of participant's movement in the 2D-plane was mapped to center frequency of the filter (50 to 1100 Hz) and to Q-factor (1.8 to 4.0). Amplitude modulation of the filtered signal was carried out using the rand~ object, with input parameter 3 Hz. Finally, velocity magnitude was logarithmically scaled to amplitude of the signal, so that no sound was heard when the participant did not move. Sound model 2 (S2) was implemented in a similar manner as S1, with the difference that the resonance filter's center frequency was set to 100–900 Hz and Q-factor range was set to 0.1–0.3. Amplitude modulation was also increased to 18 Hz. The final sound model (S3) was also based on filtering white noise, but was implemented using a band-pass filter (object biquad~). Just like for the other two sound models, velocity magnitude was mapped to the center frequency (100–3000 Hz) and Q-factor (0.01–0.6). Amplitude modulation was achieved by triggering peaks using the curve~ object^8^ which produced a non-linear ramp of length 250 ms, triggered every 50 to 800 ms, depending on velocity. See Figure 1 for the spectral content of 2 min of sound models S1, S2, and S3.

#### 3.1.4. Experimental procedure

Groups of 3–4 children were studied in each recording session. The participants were wearing hats with attached rigid body markers; trajectories could thus be defined as collections of consecutive points corresponding to the positions of the tracked head while performing a locomotor movement. We assume that head movements carry enough information about the children's expressiveness based on previous findings by Dahl and Friberg (2007) suggesting that expressive movements produced by musicians' head movements are as informative as whole body movements. Each experiment began with a brief introduction by the test leader, explaining to the participants that they were allowed to move freely in the motion capture area of the room (something that the younger participant group failed to do, thereby causing irregular data and problems with loss of tracking), and that their movements would produce sounds. Instructions were read from a pre-written manuscript. The instructions were followed by the music condition M1, in which the participants were allowed to move freely to music, but did not trigger any sounds themselves. After M1, a counterbalanced order of the sound models S1–S3 was presented to the participants. For these sound model conditions, participant's rigid body markers were mapped so that movement triggered sounds. Each sound model was presented six times. The entire experiment ended with another music model, namely M2, which was not either mapped to movement of the participants. The music conditions were 60 s long; the sound model conditions S1–S3 were 36 s long. Each experimental session lasted approximately 13.5 min. Since each group participated in two sessions of the experiment, each sound model was presented 12 times in total, resulting in 12 observations per sound model and participant, respectively.

#### 3.1.5. Analysis of movement features

The longitudinal data collected for the three level repeated measures experiment resulted in a data set in which repeated measurements (level 1) were nested within in our unit of analysis, i.e., participants (level 2), which were in turn nested within experiment groups (level 3). We used R (R Core Team, 2014) and the lme4 package (Bates et al., 2015) to perform a linear mixed model (LMM) analysis of the relationship between movement features and sound model. Linear mixed-effects models are an extension of linear regression models for data that is collected in groups. Numerous studies have demonstrated the advantages of mixed effect models over traditional random-effects ANOVAs (e.g., Baayen et al., 2008; Quené and van den Bergh, 2008). A mixed-effect model consists of fixed (FE) and random effects (RE), where FE are the predictors and RE are associated with experimental units on an individual level, drawn at random from a population.

The standard form of a linear mixed-effect model is defined in Equation (7), where *y* is the known response variable, *X* is a fixed-effects design matrix, β is an unknown fixed-effect vector containing the regression coefficients, *Z* is a random-effects design matrix, *b* is an unknown random-effects vector and ϵ is the unknown observation error vector.

(7) $$\begin{array}{l} y=X\beta +Zb+\epsilon \end{array}$$

Our main goal was to determine which predictors that were statistically significant and how changes in the predictors relate to changes in the response variable, not to build a model that could exactly emulate the effect of sonification on participant behavior. Since our research interest is centered around understanding why mean values of the dependent variable vary, we focused mainly on defining random intercept models. We defined a random intercept model for each feature index (*FI*) according to Equation (1) in which feature magnitude was as function of the fixed effect of sound model. A time variable (observation number 1–12) and session factor (recording before or after lunch) was also added as fixed effects when these were found to be significant, see Equation (2). The model resolved for non-independence by assuming different random intercepts for each participant and group, respectively. More complicated designs were also investigated, however, no random slope models converged. We described model fit by using the marginal and conditional *R*^2^ for mixed-effects models, obtained using the r.squaredGLMM function in version 1.10.0 of the MuMIn package in R (Nakagawa and Schielzeth, 2013; Johnson, 2014; Barto, 2016). The marginal *R*^2^ describes the proportion of variation explained by fixed effects and the conditional *R*^2^ describes the proportion of variation in the data explained by both fixed and random effects (Nakagawa and Schielzeth, 2013).

(8) FI~ sound model+(1|group)+(1|participant)+ϵ

(9) $$\begin{array}{l} FI\sim \;sound\;model+session+observation\\ +(1|group)+(1|participant)+\epsilon \end{array}$$

### 3.2. Results

**Figure 2:**
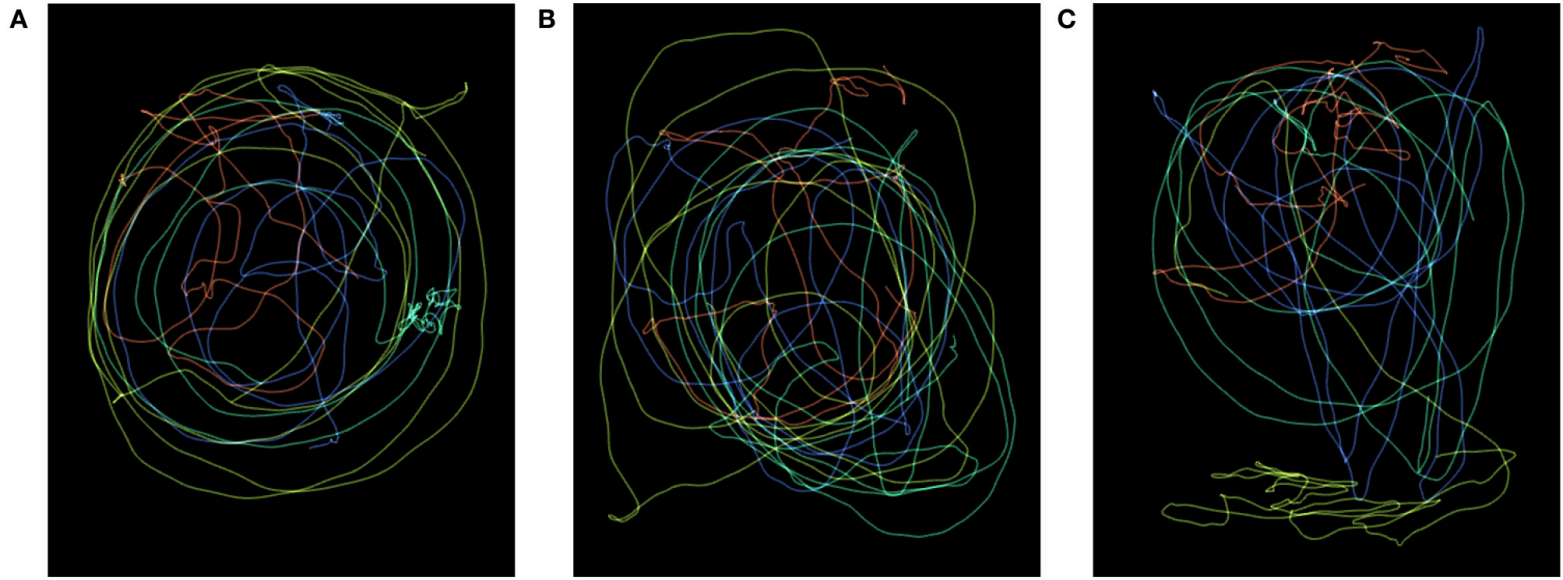
**Heat maps**. Example of the trajectories performed by the four children in a group, for the three different sound models S1–S3. Each color represents one child, brighter colors corresponds to higher velocity. **(A)** Sound model S1. **(B)** Sound model S2. **(C)** Sound model S3.

Examples of trajectories performed by a group of children for the three sound models are seen in Figure 2. Initial inspection of the data indicated considerable inter-participant variability. Issues with crossover effects (i.e., that one marker was accidentally mistaken for another marker number so that a swap of trajectories occurred for specific participants) and occlusion effects (resulting in gaps in the data) were identified in the initial stage of the data analysis. Since our analysis approach was based on computing higher order derivatives, we decided to remove all observations where crossover effects occurred, so as to reduce the risk of undesired peaks in the computed features. Observations in which tracking was insufficient due to occlusion or contained too few data points (due to the fact that participants moved outside of the trackable area) were also removed.

The recorded movement data was trimmed to 25 s long excerpts per observation, removing the first and last 6 s (original observations were 36 s long + 1 s fade between sound models). Trimming was done in order to include only the middle part of each observation. This was done to ensure that the transitions that contained fading between sound models were not included in the analysis. Moreover, trimming was done to ensure that children had stabilized their movement pattern for the sound model that was currently presented and had been active for at least a time interval of 6 s. One observation was thus defined as a recording segment of 25 s, for one specific participant and sound model. Two-dimensional tracked movement data was thereafter used to calculate the following features for all 25-s excerpts: Energy Index (*EI*), Smoothness Index (*SI*) and Directness Index (*DI*).

**Figure 3:**
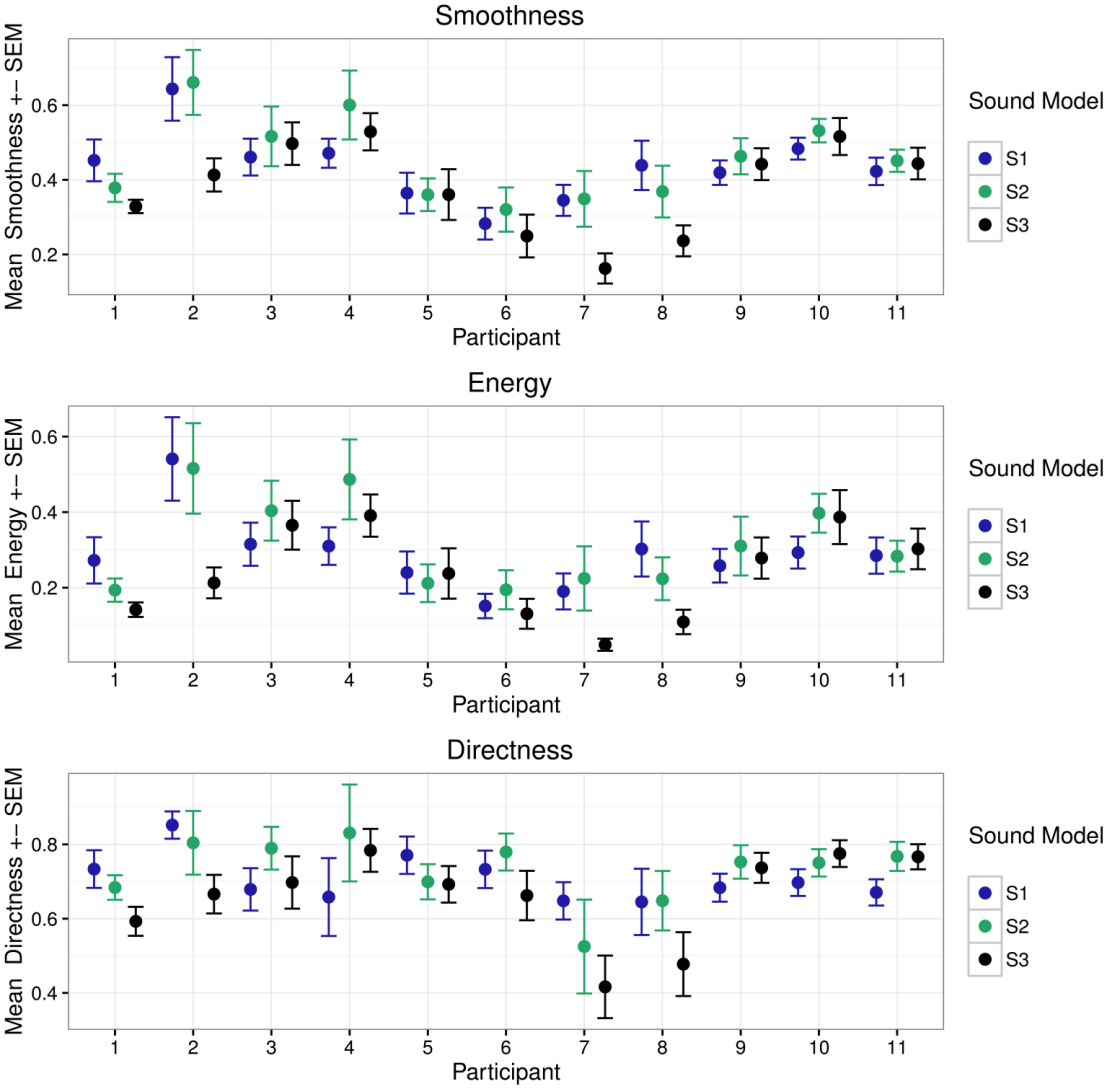
**Mean metrics per participant for the three sound models (*n* = 12 observations for each model)**. P1–P4 belong to group 1, P5–P8 belong to group 2, and P9–P11 belong to group 3.

Mean values were computed for all recording segments, resulting in 12 observations per participant and sound model (six observations from the experimental session taking place before lunch and six observations from the experimental session after lunch). Data was then normalized to the range of 0 to 1. After removal of observations with erroneous tracking, we obtained a total of 302 observations (total number before removal was 396). A summary of the computed metrics per sound model and participant can be seen in Figure 3.

#### 3.2.1. Energy index

**Figure 4:**
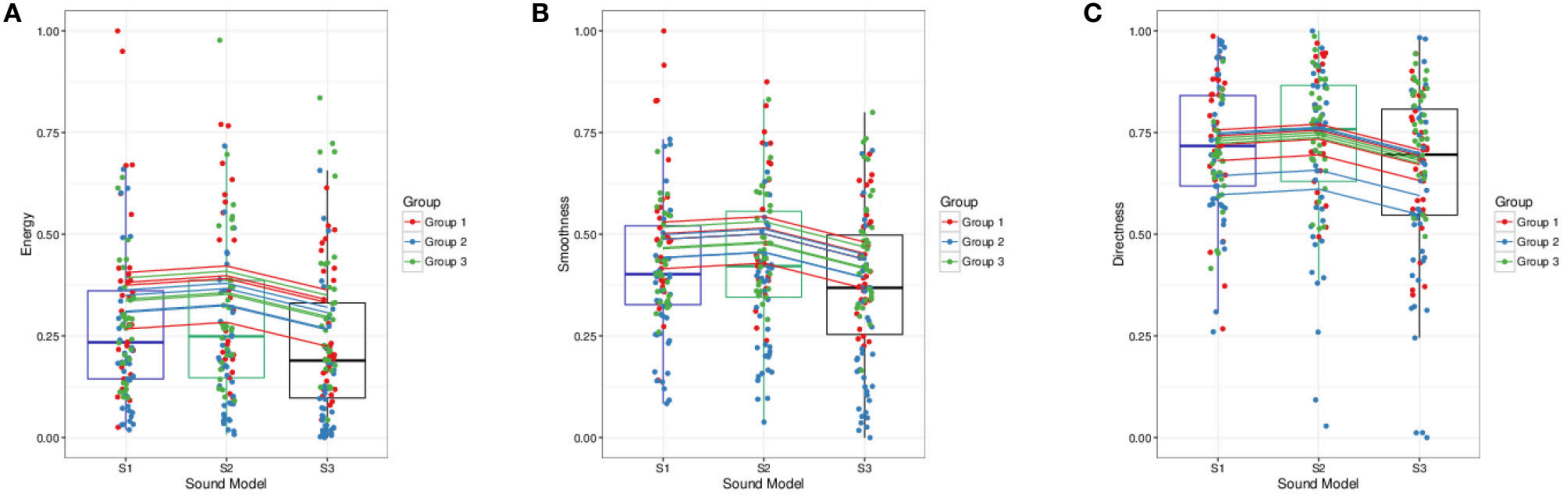
**Fitted (points, boxplots) vs. predicted values (lines) for movement features**. **(A)** Energy Index *EI*. **(B)** Smoothness Index SI. **(C)** Directness Index DI.

Descriptive statistics per sound model is seen in Table 1 (results obtained when collapsing all observations). A LMM analysis of the relationship between sound model and Energy Index *EI* was carried out according to the formula specified in Equation (2). Visual inspection of residual plots did not reveal any obvious deviations from normality. *P*-values were obtained by likelihood ratio tests of the full model with the effect in question against the null model without the effect in question. Sound model significantly affected energy, $\chi_{\left(1\right)}^{2}=7.593,p=0.022$. Recording session and observation number also significantly affected *EI*; $\chi_{\left(2\right)}^{2}\;=\;3.855,p=0.050$, lowering *EI* by 0.043±0.023 by session, and $\chi_{\left(3\right)}^{2}=22.989,p=1.630e-06$, lowering *EI* by 0.009 ± 0.002 by observation number. Afternoon sessions had generally lower *EI* values. *EI* also decreased for an increasing number of observations. This could possibly be explained by fatigue. Tukey's method for multiple comparisons of means indicated a significant difference between S2 and S3 (*p* = 0.033). Estimate for difference between S2 and S3 was −0.059±0.024. Although not significant (*p* = 0.163), estimate for difference between S1 and S3 were −0.043±0.023. Standard deviation described by random effects for participants, groups and residuals were 0.052, 0.054, and 0.168, respectively. The high value for residual standard deviation could well indicate that there might be effects that the model does not account for. Using a simple intercept model as the one defined in Equation (7) and computing pseudo-R-square including only sound model as fixed factor, sonification could be said to explain about 2.069% of the variabililty in *EI*. The entire model defined in Equation (2) accounted for a total of 24.284% in *EI* variability. A summary of predicted energy values, involving both fixed and random effects for sound model, is seen in Figure 4A.

**Table 1:** **Descriptive statistics for Energy Index *EI*, Smoothness Index *SI* and Directness Index *DI***.

	**Mean**	**Median**	**SD**	***N***	**SEM**
**Energy index**					
S1	0.279	0.235	0.191	99	0.019
S2	0.291	0.249	0.199	97	0.020
S3	0.231	0.189	0.188	106	0.018
	**Mean**	**Median**	**SD**	***N***	**SEM**
**Smoothness index**					
S1	0.426	0.402	0.171	99	0.017
S2	0.434	0.422	0.175	97	0.018
S3	0.374	0.368	0.185	106	0.018
	**Mean**	**Median**	**SD**	***N***	**SEM**
**Directness index**					
S1	0.711	0.717	0.161	99	0.0162
S2	0.724	0.759	0.184	97	0.0187
S3	0.662	0.696	0.201	106	0.0196

#### 3.2.2. Smoothness index

Analysis of the relationship between sound model and Smoothness Index *SI* was carried out according to the method described for Energy Index *EI*. Sound model significantly affected smoothness SI, $\chi_{\left(1\right)}^{2}=10.714,p=0.005$. There was also a significant effect of recording session, $\chi_{\left(2\right)}^{2}=4.424,p=0.035$, and for observation number, $\chi_{\left(3\right)}^{2}=16.819,p=4.113e-05$. Afternoon session had generally lower *SI* values; *SI* was predicted to be lowered by 0.037±0.017 between recording session 1 and 2. An increase in observation number decreased smoothness by 0.007±0.002. Tukey's method for multiple comparisons of means indicated a significant difference between S2 and S3 (*p* = 0.008). Estimate for difference between S2 and S3 was −0.063±0.021, i.e., lower smoothness for sound model S3. Although not significant *p* = 0.050, estimate for difference between S1 and S3 was −0.049±0.021. Standard deviations for the random effect of participant, group and residuals were 0.054, 0.068, and 0.149, respectively. Results obtained from computation of pseudo-R-square indicated that 2.699% of the total variability in smoothness could be described by the fixed factor. If including both session and observation number as fixed factors, as in Equation (2), the model accounts for 29.050% of the total variability. A plot of the predicted values for each participant and group, i.e., the sum of random and fixed effects coefficients for the main explanatory variable “(sound model),” can be seen in Figure 4B. Attempts to model higher level models involving random slopes converged but were not significantly different from the random intercept model defined in Equation (7).

#### 3.2.3. Directness index

Analysis of the relationship between sound model and Directness Index *DI* was carried out according to the method described for the other two indexes. Sound model significantly affected directness, $\chi_{\left(1\right)}^{2}=7.418,p=0.025$. No significant effect of observation number or session could be found, so these variables were not included in the model. Tukey's method for multiple comparisons of means indicated a significant difference between S2 and S3 (*p* = 0.026). Estimate for difference between S2 and S3 was −0.0628±0.024. Although not significant (*p* = 0.1061), estimate for difference between S1 and S3 was −0.0489 ± 0.024. Standard deviation described by random effects for participants, groups and residuals were 0.0583, 0.026, and 0.172, respectively. Sonification could be said to explain about 2.153% of the variabililty in *DI*. The model in Equation (2) accounted for a total of 13.983% in directness variability. A summarizing figure of predicted directness values involving both fixed and random effects for sound model can be seen in Figure 4C.

### 3.3. Discussion

Analysis of movement features indicate some significant differences between sound models. However, due to large inter-participant variability, the effect of sound model appears to be rather small. Nevertheless, we can see tendencies toward greater mean and median values for Smoothness Index *SI* for model S1 and S2 than for model S3. The same tendency can be found for both Energy Index *EI* and the Directness Index *DI*; mean and median values are greater for S1 and S2, compared to mean and median values for S3. Assuming that participants were moving in a more continuous manner for S1 and S2, the relatively low mean and median values for S3 might be explained by many sudden interruptions in the trajectory path. As seen in Figure 3, participants appear to show similar behavior within groups (P1–P4 belong to group 1, P5–P8 belong to group 2, P9–P11 belong to group 3).

Previous studies have proven evidence that interacting individuals can coordinate their movements through detection of visual movement information (Schmidt et al., 1990) and that visually mediated interpersonal coordination is governed by an entrainment process (Richardson et al., 2007). It is reasonable to expect the movement behaviors of the children to spread within the group, this being a result of either entrainment or conscious and unconscious social interaction. Furthermore, non-spontaneous movements were also introduced via rule-based games or free play, especially during musical condition M2 that was a piece known by the children. None of the above mentioned effects were explicitly measured in this experiment; laying bare the layered subtleties of the children's group play and interpersonal coordination patterns was well beyond the scope of this study.

## 4. Study 2: perceptual rating of audio and video

### 4.1. Method

The second study focused on investigating if sound models used in Study 1 could communicate certain hypothesized movement qualities. We therefore ran a perceptual test in which sound generated by children in the previous experiment were rated by listeners along six different perceptual scales. The test was run during the *Festival della Scienza in Genova*, (October, 27th 2015).

#### 4.1.1. Participants

Eight participants took part in the experiment, but only seven of them (5 women) completed the experiment and could therefore be included in the final analysis. The average age of these seven participants was 27.6 years (SD 11.8).

The research presented no risk to harm subjects and involved no procedures for which written consent is normally required outside of the research context. Each subject voluntarily decided to participate in the experiment and the collected data could not be coupled to the specific participant; there was no risk for potential harm resulting from a breach of confidentiality.

#### 4.1.2. Stimuli

Recorded movements and sounds from Study 1 were used to produce the stimuli. Stimuli were presented in random order to the participants and were of three conditions: videos with audio (audio-video), videos without audio (video-only), and audio only (audio-only). The sounds used corresponded to excerpts of sounds generated using S1, S2, S3, and M2. The audio-video stimuli^9^ presented movements in the horizontal plane generated by children when moving to a specific sound model and the corresponding generated sound; each participant produced a trajectory corresponding to the changing position of the head as seen in a two-dimensional plane, parallel to the floor. Videos showed dots of different colors moving on a black background, each dot representing the movements of the head of each child in a group. For the video-only stimuli the audio track was muted, and for the audio-only stimuli the video was removed. In order to provide an idea of the movements showed by the dots, heat maps of the trajectories for each of the three different sound models S1–S3 as performed by the four children in a group are presented in Figure 2.

For each of the three conditions (audio-video, video-only, audio-only) there were 12 stimuli, corresponding to 4 sound models × 3 variations. Participants were thus presented with a total of 36 stimuli. Each stimulus was 20 s long. All excerpts were taken from the first group of participants (4 children) from the morning session in Study 1, in which each sound model was presented six times. We chose to include the recordings corresponding to the first three of these variations in the current Study.

#### 4.1.3. Equipment

Stimuli were presented using an online platform^10^ and evaluated using portable tablets. All participants wore headphones^11^.

#### 4.1.4. Experimental procedure

The participants were presented with the following instructions on the screen of the tablet:

*In this test you will watch videos and listen to sounds. You will be asked to rate different properties of each of them by using sliders on the screen. Take all the necessary time, but try to answer as quickly as possible and to use the entire scale of the sliders. There is not right or wrong answer. You can repeat the playback of videos and sounds as many times as you need*.

Participants were asked to rate the stimuli along six continuous semantic differential scales describing movement quality (Fluid, Energetic, Impulsive, Fast, Expressive, and Rigid) ranging from *Not at all* to *Very much* as minimum and maximum values, respectively (e.g., not at all fast, very fast)^12^. The slider's start position was always placed in the middle of the scale, corresponding to value 50. Numerical values of the sliders were not visible to participants.

The six semantic scales were identified in previous research in which they were used in body motion analysis (for Fluid, Energetic, Impulsive, Fast, and Rigid; see Camurri et al., 2016) and for rating expressiveness in music performance resembling biological motion (for Expressive; see Juslin et al., 2002).

### 4.2. Results

The duration of the experiment was 44 min, on average (SD = 10). The participants' mean ratings were analyzed using a three-way repeated measures ANOVA, with the factors sound model (4 levels), sound model variation (3 levels), and condition (3 levels). The analysis was done separately for each of the six semantic differential scales. Before running the three-way ANOVA, a Mauchly test was run to verify if the assumption of sphericity had been met for the factors sound model, sound model variation, and condition. When needed, we report corrected degrees of freedom (using Greenhousee-Geisser estimates of sphericity). The analysis for the sound model factor is summarized below and in Table 2:

**Table 2:** **Mean ratings and effect of sound model for the six different semantical scales used in Study 2**.

	**S1**		**S2**		**S3**		**M2**	
	**Mean**	**SE**	**Mean**	**SE**	**Mean**	**SE**	**Mean**	**SE**
Energetic	55.063	6.177	46.810	6.599	36.841	5.873	63.952	3.630
Expressive	55.063^a^**^^	6.717	46.810	6.599	36.841^a^**^^^,^^b^*^^	5.873	63.952^b^*^^	3.630
Fast	46.905	4.209	59.952	5.325	52.175	6.258	48.921	4.836
Fluid	56.302^c^*^^^,^^d^*^^	6.197	47.937^c^*^^	4.262	31.222^d^*^^^,^^e^**^^	3.981	55.222^e^**^^	2.217
Impulsive	56.683	3.210	52.254	3.095	56.508	5.791	44.905	4.879
Rigid	33.048	3.435	35.413	4.851	55.952	7.438	33.905	4.028

*Significance levels*:

* *p ≤ 0.05*,

** *p ≤ 0.01*.

a *significant difference in means between S1 and S3*.

b *significant difference in means between S3 and M2*.

c *significant difference in means between S1 and S2*.

d *significant difference in means between S1 and S3*.

e *significant difference in means between S3 and M2*.

*Energetic*: There was a significant main effect of condition, *F*_(2, 12)_ = 13.609, *p* = 0.001. Stimuli presented in the audio-only condition were in general rated as more energetic than stimuli with video, i.e., stimuli in both audio-video and video-only conditions. A Bonferroni *post hoc* comparison showed that the mean Energetic rating for the audio-only condition was significantly different (higher) from that of the two other stimuli categories (*p* < 0.037). It can also be observed that stimuli with sounds S1 and M2 were rated as more energetic than the other stimuli.

*Expressive*: A significant main effect of sound model was observed [*F*_(3, 18)_ = 11.913, *p* < 0.0001]; stimuli produced using sound model S1 and M2 were rated as the most expressive ones. M2 was rated as more expressive than S1. Sound models S2 and S3 received a mean rating below 50. Sound S3 was rated significantly different from sounds S1 and M2 (Bonferroni *post hoc* comparison, *p* < 0.025). A significant interaction between condition and sound was also observed [*F*_(6, 36)_ = 5.941, *p* < 0.0001]; stimuli using sound model S1 were rated as more expressive than stimuli produced with S2 and S3 for all conditions; stimuli produced using S3 were rated as the least expressive ones for all conditions. Stimuli generated using M2 were rated as the most expressive ones for the audio-only and audio-video condition.

*Fast*: No statistically significant effects were found. Nevertheless, stimuli in the condition audio-only were on average rated as more than 60% faster than stimuli in other conditions. Stimuli corresponding to sound S1 were perceived as the slowest, while those corresponding to sound model S2 were rated as the fastest ones (about 20% faster than the other sounds).

*Fluid*: For this scale a significant effect of factor sound model was found [*F*_(1.606, 9.638)_ = 9.277, *p* = 0.007, with corrected degrees of freedom and *p*-value]. Stimuli corresponding to S1 and M2 were rated as about 30% more fluid than stimuli from S2 and S3. Stimuli from all conditions corresponding to sound model S3 were rated as significantly less fluid than other stimuli. Pairwise comparisons between all sounds showed that there was a significant mean difference between ratings for sound model S1 compared to sound models S2 and S3, as well as between sound models S3 and M2 (Bonferroni *post hoc* comparison, *p* < 0.05). A significant interaction between factors condition and sound model was also found [*F*_(1.606, 9.638)_ = 9.277, *p* = 0.007, with corrected degrees of freedom and *p*-value]. Audio-only stimuli corresponding to S2 and M2 were rated about 3 times more fluid than the other stimuli; for the video condition this difference was not present for model S2, while stimuli corresponding to sound S3 were rated as the least fluid ones in all conditions.

*Impulsive*: A significant effect of condition was found [*F*_(2, 12)_ = 6.152, *p* = 0.014]. Stimuli in the audio-only condition were in general rated as more impulsive than stimuli in the audio-video condition (Bonferroni *post hoc* comparison, *p* < 0.028). A significant interaction between factors sound and sound variation was also found [*F*_(3, 36)_ = 3.805, *p* = 0.005]. Sounds S2 and S3 were rated as the more impulsive ones in the audio-only condition. Stimuli of sound M2 were rated as the least impulsive ones.

*Rigid*: No statistically significant effect of main factors was found. However, it was observed that sound S3 was rated as the most rigid one, about 60% more rigid than the other sounds. A significant interaction between condition and sound model was also observed [*F*_(6, 36)_ = 10.42, *p* < 0.0001]. S3 was rated as the most rigid in all categories. Stimuli presented with sound S2 were rated as more rigid when presented without video, and less rigid when presented in other conditions. Stimuli including S1 and M2 received low mean ratings for all conditions and could thereby be considered to be perceived as non-rigid.

### 4.3. Discussion

To summarize, a significant main effect of sound model was observed for the scales Expressive and Fluid, in which sound models S1 and M2 were rated as more expressive and fluid than the other sound models. Sound model S3 was rated as more rigid and fast than other sound models, although this difference was not significant. A significant effect of condition was observed for scales Energetic and Impulsive. The interaction effect between condition and sound model was also observed to be significant for scales Expressive, Fluid and Rigid. These results confirm our initial hypothesis that sound model S1 would communicate the sensation of being more fluid, smoother (and possibly also slower and less rigid) while sound model S3 would be perceived as less fluid (and possibly also faster and more rigid).

## 5. Study 3: perceptual rating of sound visualizations

### 5.1. Method

We hypothesize that the properties of the body motion used by the children for generating sounds S1–S3 can be found also in abstract representations of sound, i.e., sound visualizations in the form of drawings. More specifically, our hypothesis is that there is a consistent mapping of body motion qualities from one modality (sound) to another one (sound visualizations). To investigate this, we ran a three alternative forced-choice experiment (3AFC) designed to see if participants could correctly match recordings of one sound model to an abstract visual representation (i.e., a drawing) of the same sound model.

#### 5.1.1. Participants

146 students (68 women) from the Media Technology programme at KTH took part in the experiment. Their average age was 22.4 years (SD = 2.7).

As for the previous online experiment (Study 2), the research presented no risk to harm subjects and involved no procedures for which written consent is normally required outside of the research context. Each subject voluntarily decided to participate in the online study and there was no risk for potential harm resulting from a breach of confidentiality.

#### 5.1.2. Stimuli

The 11 children who had participated in Study 1 (see Section 3 and 3.1.1 for ethics considerations) took part in a follow-up study that was set up as a drawing exercise. The children listened to excerpts of the two classical music stimuli (M1–M2) and the sonification sounds (S1–S3) that they had produced in the motion capture experiment. The excerpts were 2 min long. The children were asked to freely draw whatever they wanted while listening to each of the 2-min long five audio stimuli (S1–S3 and M1–M2). We consider these drawings to be abstract representations of the presented sounds. The idea of using drawings to depict sounds was inspired by previous work by Merer and colleagues (2008; 2013).

**Figure 5:**
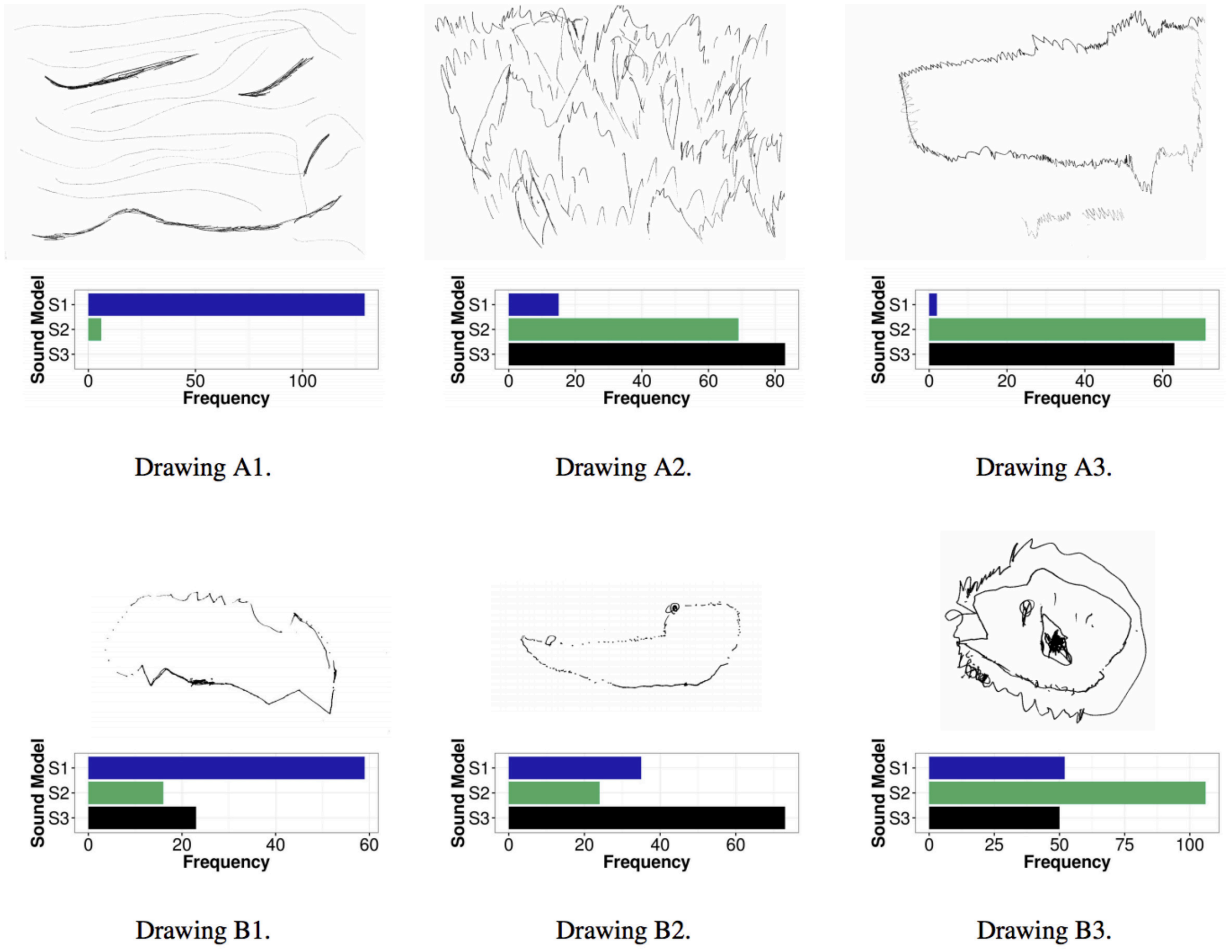
**Drawings by one 6-year girl (child A, top row) and by a 6-year boy (child B, bottom row) used in the experiment in Study 3; left to right drawings made while listening to sounds produced using model S1, S2, and S3, respectively**.

A selection of sound visualizations in the form of drawings from the drawing exercise described above was used as stimuli in the 3AFC experiment. We selected drawings that included abstract representations of the sounds from 4 children. This selection was done in order to avoid symbolic representations of the sounds (such as e.g., plants, birds or people), which could bias the perceptual ratings. Three drawings per child were used as stimuli in the 3AFC experiment: each drawing corresponded to each of sound models S1, S2, and S3^13^. Each drawing was presented with the same recorded sounds that had been presented in the drawing exercise. The drawings were processed to be black and white to enable the participants to focus simply on the patterns and trajectories in the drawings, not on color properties (see Figure 5).

#### 5.1.3. Equipment

Stimuli were presented using the same online platform as in Study 2^14^. A link to the experiment ^15^ was sent via email to the participants, who could use their preferred device (computer or portable device) to participate in the experiment.

### 5.2. Experimental procedure

The following instructions were given to the participants:

*In this test you will be asked to connect sounds to drawings. Take all the necessary time, but try to answer as quickly as possible. There is not right or wrong answer. You can repeat the playback of sounds as many times as you need. You are allowed to answer to the questions while the sound is still playing*.

The total number of stimuli consisted 4 sets of drawings (from 4 different participants) × 3 sound models, giving a total of 12 stimuli. Stimuli were presented in a randomized order per set of drawings. Participants were asked to make a three-alternative forced choice (3AFC) between three drawings and the presented sound.

### 5.3. Results

Based on findings from Study 1 in which it was concluded that results from the youngest children should be excluded from the analysis since these participants did not follow instructions correctly and also not fully understood the experimental task (see Section 3.1.1), drawings produced by the youngest children were excluded from the analysis. This decision was done in order to follow the same methodology as the one used in Study 1. Analysis of the obtained results were thus done on the answers obtained for the 2 drawings that had been produced by the oldest children (one girl and one boy; referred to as child A and child B in Figure 5).

We ran a chi-square test to analyze the association between the two variables sound model (S1–S3) and drawing [$\chi_{\left(df=10,N=876\right)}^{2}=436.514,p=0.000$]. The results indicated a significant association between the two variables (expected counts were greater than 5), thus implying that certain sound models were associated to certain drawings. In particular, sound model S1 was clearly associated to drawings of S1, while S2 and S3 were mostly associated to drawings of either one of these two sound models (see Figure 5 for more details).

**Figure 6:**
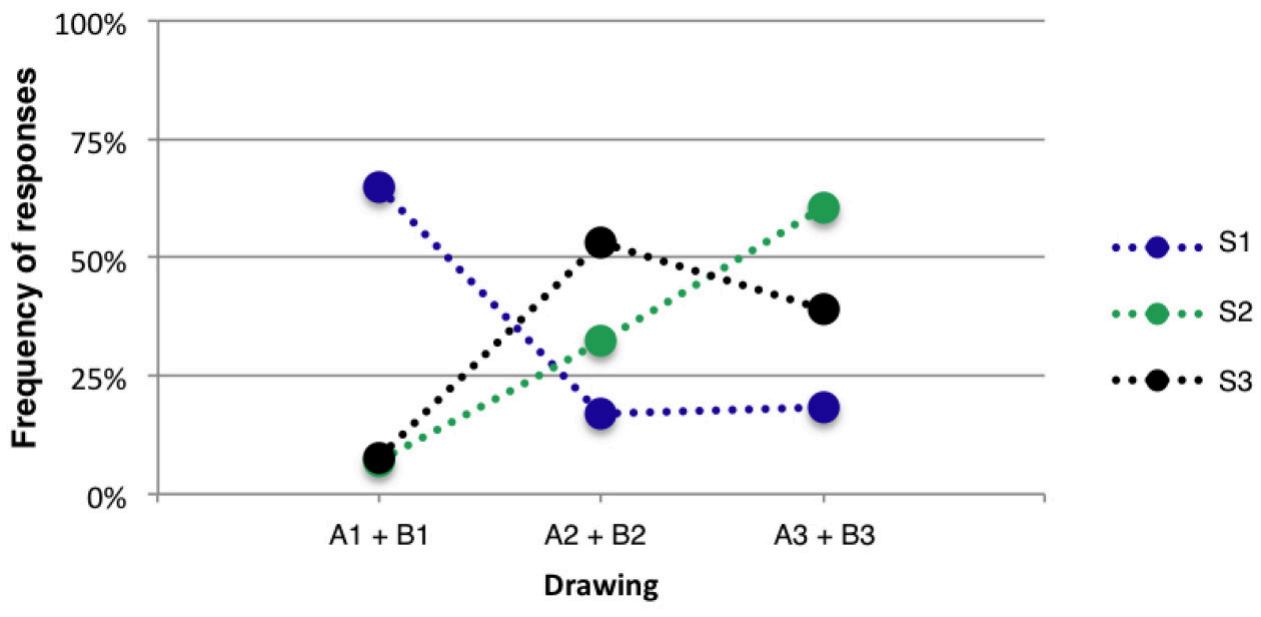
**Association between sounds produced using S1, S2 and S3, and drawings (see Figure 5)**.

Analysis of response frequency when collapsing all results per drawing class (i.e., which sound model the drawing was actually depicting) showed that 64% of the participants associated sound model S1 to the corresponding visual representation of sound model S1. Only 8% of the participants associated sound model S1 to drawings depicting sound model S2 or S3 (see Figure 6).

### 5.4. Discussion

As mentioned by Glette et al. (2010), participants associations to sounds are very subjective and tracings of sound can therefore vary a lot between participants. Nevertheless, results from the sound visualization experiment indicated that participants rather easily identified drawings portraying sound model S1. Drawings of sound model S2 and S3 did also match to the correct sound model for child A. For child B we can see an opposite behavior in which drawing of sound model S2 was matched to sound model S3, and vice versa. These results confirmed our hypothesis that qualities of movement present in the sound recordings could be transferred into sound visualizations produced by children and that listeners could subsequently recognize these qualities.

## 6. General discussion

Analysis of movement features in Study 1 indicated significant effect of sound model. However, due to large inter-participant variability, the effect of sound model appeared to be rather small. In general, the effect of group belonging appears to also be important in this context, as well as aspects of fatigue (observed in terms of significant effect of observation number and session number). The children showed different behavior throughout the experimental sessions and moved in a manner that was not very consistent; their movement patterns appeared to be more guided by the social interaction with other children than the overall features of the sounds. Nevertheless, we can see some tendencies toward greater mean and median values of smoothness and directness for the sound models S1 and S2 than for sound model S3. This might indicate that there are aspects related to sound model which would be interesting to explore further in the context of spontaneous movement induced by interactive sonification. Considering the open structure of the experiment (the children were allowed to move freely and interact with each other in groups), it is likely that a more controlled experiment would provide clearer results with higher statistical power. The fact that the children were very young and behaved accordingly was of course an aspect that affected the results (as previously mentioned, some of the data had to be excluded from the analysis). We propose follow-up studies in which the same sonification models are evaluated in a more controlled setting to fully be able to evaluate the effects of sonification model on induced movement.

Findings from the perceptual rating experiment (Study 2) indicate a significant effect of sound model on the perception of expressiveness and fluidity. More precisely, sound model S1 was found to communicate the sensation of being more fluid when compared to sound model S3. Although not significant, S3 was rated as 60% more rigid and fast than other sound models. One could suggest that certain properties of sound model S1 results in the fact that sounds produced using this model are perceived as more fluid and slow than sounds produced using sound model S3. Interestingly, we could also detect significant interactions between sound model and condition (audio-only, video-only or audio-video) for the expressiveness-, fluidity- and rigidity scales. These results support the hypothesis that different sound models can, by themselves, be perceived differently, but also that perception of movement qualities is indeed a multimodal phenomenon. Interestingly, the effect of condition was significant for energy- and impulsivity scales: when stimuli were only auditory it was perceived as more energetic and more impulsive than when stimuli also included a video visualization counterpart. This confirms the ability of sound to communicate high-level qualities of movement.

Although the experimental methodology of Study 3 could have been simplified, for example by using simple sound visualizations containing caricatures similar to the ones in the *Bouba-Kiki* experiments by Kohler (1929, 1947) instead of drawings, the ability to communicate high-level qualitative features of movement using only sound as a medium could be confirmed in the study. The qualities of a movement present in audio recordings were recognized in sound visualizations produced by children. Drawings portraying sound model S1 were rather easily identified as being a portrayal of the actual sound model S1. This supports our hypothesis that certain qualities of movement present in sound recordings can actually be translated into sound visualizations (and that these sound visualizations subsequently can be recognized by another independent group of listeners). Similarly to what Merer et al. (2013) suggested, i.e., that drawings are a relevant means of describing motion in an intuitive way, we can conclude that drawings can be successfully used as a tool for describing movement features which are present in a sound through meaningful sonification of movement properties.

To conclude, the three studies presented in this paper suggest that sound models can be designed and controlled so that: (1) sound might have an effect on bodily movement characteristics; (2) different sounds can be associated with different levels of motion qualities (e.g., fluid and expressive); (3) sound-only stimuli can evoke stronger perceived properties of movement (e.g., energetic, impulsive) compared to video stimuli; (4) sounds generated by body motion can be represented and associated with sound visualizations (drawings) in a meaningful way. The results obtained support the existence of a cross-modal mapping of body motion qualities from bodily movement to sounds and the potential of using interactive sonification to communicate high-level features of human movement data. Sound can be translated and understood from bodily motion, conveyed through sound visualizations in the form of drawings, and translated back from sound visualizations to sound.

## Author contributions

RB: supervised the project; EF, RB, and LE: designed and performed the experiments; LE developed the software used for communication; EF developed sound models and analyzed collected data from study 1 as well as edited the paper; RB analyzed data from study 2 and 3; PA developed analytical tools using the *EyesWeb* software.

## Funding

This research has received funding from the European Unions Horizon 2020 research and innovation programme under grant agreement No 6455533 (DANCE) 2. DANCE investigates how affective and relational qualities of body movement can be expressed, represented, and analyzed by the auditory channel.

### Conflict of interest statement

The authors declare that the research was conducted in the absence of any commercial or financial relationships that could be construed as a potential conflict of interest.

## Footnotes

^1^For the management of participants' personal data, we followed rules according the KTH Royal Institute of Technologys Ethics Officer (Personuppgiftsombud).

^2^Optitrack Prime 41: https://www.optitrack.com/products/prime-41/

^3^Optitrack Motive: https://www.optitrack.com/products/motive/

^4^NatNet SDK: https://www.optitrack.com/products/natnet-sdk/

^5^Open Sound Control: http://opensoundcontrol.org

^6^Max/MSP: https://cycling74.com/products/max/

^7^EyesWeb XMI platform http://www.infomus.org/eyesweb_ita.php

^8^connected to the following message box: “0.85, 0.0 50 0.5 0.0 200 −0.5”

^9^Examples of stimuli can be found here: https://kth.box.com/s/818vbkb6m6nlkgk4vb4wvt0zt52y5uqy

^10^SurveyGizmo: http://surveygizmo.com

^11^The test can be found at: http://www.surveygizmo.com/s3/2396910/Evaluation-of-sound-and-video-qualities

^12^Depending if the stimulus was a video or a sound only, the question to experiment participants was “*The sound reminds of a movement that is:”* or “*The movements of the dots are:”*, respectively. Each scale was varying between 1 and 100.

^13^Drawings of the classical sounds were not included as stimuli in the experiment, since the research hypothesis of Study 3 only addressed aspects of the sonification models, and not music stimuli in general.

^14^SurveyGizmo: http://surveygizmo.com

^15^The test can be found and performed at the following link: http://www.surveygizmo.com/s3/2607938/e9087ebd3a82
